# Early Intervention of *Elateriospermum tapos* Yoghurt in Obese Dams Mitigates Intergenerational Cognitive Deficits and Thigmotactic Behaviour in Male Offspring via the Modulation of Metabolic Profile

**DOI:** 10.3390/nu15061523

**Published:** 2023-03-21

**Authors:** Ruth Naomi, Rusydatul Nabila Mahmad Rusli, Teoh Soo Huat, Hashim Embong, Hasnah Bahari, Mohd Amir Kamaruzzaman

**Affiliations:** 1 Department of Human Anatomy, Faculty of Medicine and Health Sciences, Universiti Putra Malaysia, Serdang 43400, Malaysia; 2 Advanced Medical and Dental Institute, Universiti Sains Malaysia, Penang 13200, Malaysia; 3 Department of Emergency Medicine, Faculty of Medicine, Universiti Kebangsaan Malaysia, Kuala Lumpur 50300, Malaysia; 4 Department of Anatomy, Faculty of Medicine, Universiti Kebangsaan Malaysia, Jalan Yaacob Latiff, Cheras, Kuala Lumpur 56000, Malaysia

**Keywords:** natural extract, yoghurt, maternal overnutrition, intergenerational effect, lipid profile, cognition, anxiety

## Abstract

Maternal obesity is an intergenerational vicious cycle and one of the primary causes of cognitive deficits and high anxiety levels in offspring, which often manifest independently of sex. It is proven that curbing the intergenerational inheritance of obesity through early intervention during the gestation period has a positive outcome on the body composition, cognitive function, and anxiety level of the offspring. A recent discovery shows that the consumption of *Elateriospermum tapos* (*E. tapos*) seed extract modulates body mass and ameliorates stress hormones in obese dams, while a probiotic bacterial strain can cross the placenta and boost a child’s memory. Thus, we speculate that probiotics are the best medium to integrate plant extract (*E. tapos* extract) to access the effect on the child’s cognition. Thus, this study aimed to investigate the early intervention of *E. tapos* yoghurt in obese dams in the cognition and anxiety levels of male offspring. In this study, 40 female rats were fed with a high-fat diet (HFD) to induce obesity before pregnancy, while another 8 rats were fed with standard rat pellets for 16 weeks. Upon successful copulation, treatment was initiated for the obese dams up to the postnatal day (PND) 21. The groups included normal chow and saline (NS), HFD and saline (HS), HFD and yoghurt (HY), HFD and 5 mg/kg *E. tapos* yoghurt (HYT5), HFD and 50 mg/kg *E. tapos* yoghurt (HYT50), and HFD and 500 mg/kg *E. tapos* yoghurt (HYT500). All rats were euthanised on PND 21, and the body mass index (BMI), Lee index, and waist circumference were measured for the male offspring. Hippocampal-dependent memory tests and open field tests were conducted to access for cognition and anxiety status. Fasting blood glucose (FBG), total fat (%), insulin, leptin, lipid profile, and antioxidant parameter on serum and hypothalamus (FRAP and GSH) were accessed on PND 21. The result shows male offspring of 50 mg/kg-supplemented obese dams have comparable total fat (%), lipid profile, insulin level, FBG level, plasma insulin level, recognition index, low anxiety level, and improved hypothalamic FRAP and GSH levels to the normal group. In conclusion, this study highlights that the effect of early intervention of our novel formulation of *E. tapos* yoghurt in obese dams alleviates cognitive deficits and anxiety in male offspring by modulating metabolic profiles at the dose of 50 mg/kg.

## 1. Introduction

Excessive gestational weight gain is often linked to a neurodevelopmental delay in the offspring [1]. Maternal obesity is an intergenerational vicious cycle, and the obesogenic can be passed to the next generation [2]. Maternal overnutrition in the foetus can induce leptin resistance in the hypothalamus, thereby stimulating the overexpression of appetite-regulating peptides. As such, there is a positive correlation between overnutrition and the foetus’s BMI, waist circumference, length, and adiposity [3]. Prolonged consumption of a high-fat diet (HFD) is one of the causes of prepregnancy weight gain, and the persistent intake of an HFD is directly linked to poor memory in the offspring [4]. One of the reasons for this scenario could be due to neuroinflammation in the hippocampus. Numerous in vivo studies show that the consumption of an HFD could activate glial cells, leading to neuroinflammation and eventually cognitive dysfunction. This proves that there is a strong association between lipid profile dysregulation and memory decline [5]. Aside from this, the prolonged consumption of an HFD can negatively affect peripheral organs and neuronal plasticity [6]. One possible mechanism for this is that a HFD alters the function of the mitochondria in the brain, which are responsible for providing energy to support synaptic plasticity and controlling the production of reactive oxygen species (ROS). Because synaptic areas have particularly high energy demands, the negative effects of an HFD on mitochondrial function may be more pronounced in these areas. When mitochondria in the brain become dysfunctional due to inflammation and oxidative stress, they can no longer provide enough energy to support synaptic plasticity, leading to impaired neuronal function and neurodegeneration [6,7].

Along with this, the intake of an HFD induces oxidative stress in the hypothalamus. This is evident based on the drastic reduction of enzymatic antioxidant activity, such as glutathione (GSH) [8] and nonenzymatic antioxidants, such as ferric reducing ability of plasma (FRAP) in the plasma [9]. There is growing evidence suggesting that the consumption of an HFD may have negative effects on cognitive function, both in mothers and their offspring. In animal studies, maternal consumption of an HFD during pregnancy and lactation has been shown to impair cognitive function in offspring. These offspring exhibit deficits in learning and memory tasks, as well as increased anxiety-like behaviour [10]. Various mechanisms have been identified to cause cognitive decline in the offspring of maternal subjects that consume an HFD during pregnancy and lactation. Some of them are inflammation, impaired blood–brain barrier (BBB), epigenetic modifications, and alterations in the gut microbiome. One possible explanation is chronic low-grade inflammation induced by an HFD, which can cause cellular damage and negatively affect cognitive function. Inflammatory molecules may cross the placenta and impact fetal brain development [11]. Another mechanism is the impairment of the BBB in offspring, allowing harmful substances to enter the brain and disrupt normal cognitive function [12]. Maternal HFD consumption can also induce epigenetic modifications in the offspring’s DNA, altering the expression of genes involved in brain development and function, leading to cognitive impairments [13]. In addition, maternal HFD consumption can also alter the composition of the gut microbiome in both the mother and offspring, which has been associated with cognitive impairments [14]. However, HFD-induced cognitive deficits are often exhibited in a sex-dependent manner as male offspring are more prone to develop poor memory compared to female offspring. The male offspring of obese dams often display an anxiety-like behaviour and prefer to exhibit thigmotactic behaviour [15]. So, males are more vulnerable to develop schizophrenia [16], vascular dementia [17], and attention deficit hyperactivity disorder (ADHD) [18]. Hence, there is an urgent need to curb maternal obesity-induced cognitive dysfunction and anxiety in the offspring to prevent long-term neurodevelopmental complications. The currently available treatments, such as Orlistat, is linked with adverse effects, such as acute kidney injury [19], while prolonged intake of Sibutramine can result in asthenia, amnesia, and obstipation [20].

Thusly, natural products are said to be the best and most effective option to curb obesity and its complications. In this context, our preliminary study shows that a local tropical plant known as *Elateriospermum tapos* (*E. tapos*) comprises a variety of polyphenol and bioactive molecules that can exhibit an antiobesity effect in obese dams by suppressing the activity of lipoprotein lipase [21]. Studies performed by Abidin et al., (2020) show that *E. tapos* extract can modulate stress hormones [22]. *E. tapos* is a semideciduous plant that can be found in the deep forest of Southeast Asian countries, such as Borneo, Thailand, Peninsular Malaysia, and Indonesia. The seed of the *E. tapos* plant contains high levels of proteins, unsaturated fatty acids, white oleic acids, a-linolenic acid [23], omega-3 essential fatty acids, and amygdalin, which have potential therapeutic benefits to health [24]. The seed of the plant can either be eaten raw or cooked prior to eating. Yet, the excessive consumption of *E. tapos* seed can cause dizziness [24]. On the other hand, probiotic consumption favours the body’s metabolism [25]. This is because certain probiotic strains can cross the placental barrier and exhibit beneficial effects on the foetus [26]. The safety efficacy study on *E. tapos* seed shows that it is safe for consumption up to 2000 mg/kg [27]. Thus, through a related literature search, it has been speculated that probiotics are the most effective medium to supplement the plant extract to the growing foetus [28,29,30]. Hence, this study was designed to investigate the effect of early intervention of *E. tapos* yoghurt in the male offspring of obese dams focusing on intergenerational cognitive deficits and anxiety levels.

## 2. Materials and Methods

### 2.1. Collection and Confirmation of E. tapos Seed

The *E. tapos* seed was obtained from the Forest Research Institute of Malaysia (FRIM) and was sent for identification and confirmation to Herbarium Biodiversity Unit, University Putra Malaysia (UPM) under the approval code of UPM SK 3154/17.

### 2.2. Ethanol Extraction of E. tapos Seed

Upon confirmation, about 500 g of *E. tapos* seed was soaked in 2 litre of 95% ethanol for 7 days. On the 7th day, we collected the filtrate and evaporated it using a rotary evaporator, and maltodextrin powder was added to the filtrate at a ratio of 1:1 [31] and was dried overnight in the oven at 45 °C [32]. The following day, the powder form of *E. tapos* was collected and stored in the freezer until further usage.

### 2.3. Formulation of E. tapos Yoghurt

The yoghurt was prepared by boiling 100 mL of full cream milk (Dutch Lady Purefarm UHT) at 70 °C for 20–30 min. Then, the milk was allowed to cool down at room temperature, and a starter culture consisting of live bacterial culture of *Streptococcus thermophilus* APC151 and *Lactobacillus delbrueckii subsp. Bulgaricus* ATCC 11842 was added. The mixture was then incubated in the yoghurt maker (Pensonic PYM-700) for a maximum of 8 h and refrigerated at 4 °C overnight. The following day, the preprepared *E. tapos* powder was added to the yoghurt at the ratio of 2 g per 100 mL yoghurt and stirred well [33].

### 2.4. High-Fat Diet Preparation

The preparation of a high-fat diet (HFD) was adapted from elsewhere. A total of 68% standard chow pellet (Gold Coin Feedmills (M) Sdn Bhd, Selangor, Malaysia), 6% corn oil (Vecorn, Yee Lee Corporation Berhad, Kuala Lumpur, Malaysia), 6% ghee (Crispo, Crispo-Tato (M) Sdn Bhd, Kuala Lumpur, Malaysia), and 20% milk powder (Dutch lady, Dutch Lady Milk Industries Berhad, Selangor, Malaysia) were mixed and baked at 100 °C for 1–2 h before being refrigerated overnight at 4 °C [34].

### 2.5. Experimental Animals

All animal procedures were conducted based on the guideline provided by the Institutional Animal Care and Use Committee (IACUC), UPM under the approval code of UPM/IACUC/AUP-R025/2022. In this study, 48 female Sprague–Dawley (SD) rats weighing from 150 to 200 g were used. All rats were acclimatised for one week at 12/12 light/dark cycles in a temperature-controlled room (23–24 °C). During the acclimatisation period, all rats were supplemented with standard chow pellet (Gold Coin Feedmills (M) Sdn Bhd, Selangor, Malaysia) containing 23.4% protein, 4.5% fat, and 72.1% carbohydrates with free access to water (bottle feeding) [35].

### 2.6. Obesity Induction

Female SD rats (*n* = 40) were supplemented with HFD pellets for 16 weeks, while the control group (*n* = 8) received standard chow pellets. Obesity was confirmed in HFD-supplemented group upon confirming a 13% mean body weight increase compared to control groups [36].

### 2.7. Mating, Gestation, and Weaning

Upon successful obesity induction, both the control group (*n* = 8) and obese rats (*n* = 40) proceeded with mating by placing one male rat per 2 female rats in a cage. The next morning, all female rats were subjected to manual palpation, and vaginal smears were collected and observed under a microscope for the presence of sperm. The first day for detection of sperm was recorded as postcoital day 0 [37], and treatment with different concentrations of *E. tapos* yoghurt was initiated in the obese dams. The treatment groups were as follows: normal chow and saline (NS), HFD and saline (HS), HFD and yoghurt (HY), HFD and 5 mg/kg *E. tapos* yoghurt (HYT5), HFD and 50 mg/kg *E. tapos* yoghurt (HYT50), and HFD and 500 mg/kg *E. tapos* yoghurt (HYT500). The treatment was given through oral gavage until postnatal (PND) 21 for the obese dams. No direct treatments were administered to the offspring.

### 2.8. Anthropometrical Determinations

On PND 21, all male offspring were sacrificed using carbon dioxide overdose. Body mass index (BMI), Lee index, and waist circumference were determined on the male offspring on PND 21. The waist circumference of the rats was measured in a ventral posture using a flexible tape at the greatest portion of their stomach [38]. To measure the length, the rat was positioned on a flat surface with its head and tail aligned, and then the distance between the tip of the nose and the base of the tail was measured using a ruler. To measure nose-to-anus length in rats, the same technique as measuring total length was used, but with the tail excluded. The distance was measured from the tip of the nose to the anus [39]. The length, nose-to-anus length, and waist circumference were measured 3 consecutive times (repeated measurements) to ensure intraobserver variability. From the recorded measurement, BMI and Lee index were calculated using the formula below:

(a) BMI = weight (g)/(length (cm))^2^;
  Obesity threshold: BMI > 0.687 g/cm^2^ [40].
(b) Lee index = (weight (g)/length (cm))^(1/3);
  Obesity threshold: Lee index > 310 g [41].

### 2.9. Anxiety Test

The thigmotactic behaviour in male offspring was determined through an open-field test (OFT) on PND 21. In this test, a grey PVC box (Muromachi Kikai Co., Tokyo, Japan) measuring 80 cm in width, 80 cm in length, and 50 cm in height was used. The test was conducted during the light illumination cycle. During the test, the rats were placed in one corner of the box, and the time spent close to the wall (thigmotaxis), time spent at the central region, and total distance travelled were recorded using ANY-maze™ Video Tracking System (Stoelting Co., Wood Dale, IL, USA) [42].

### 2.10. Novel Object Recognition Test (NORT) and Place Recognition Test (PRT)

The hippocampal-dependent memory tests known as NORT and PRT were performed on all male offspring on PND 21. For both these tests, all male offspring were allowed to acclimatise in a grey PVC box (Muromachi Kikai Co., Tokyo, Japan) measuring 80 cm in width, 80 cm in length, and 50 cm in height for 5 min on the first two days. On the 3rd day, for NORT, the rats were allowed to tour around an identical object (1.25-litre plastic bottle) for 5 min (trial phase) followed by a 5 min retention phase. During the testing phase, a novel object was added (mug) to the PVC box, and the rats were placed into the box. The total time spent on the novel object was recorded using ANY-maze™ Video Tracking System (Stoelting Co., Wood Dale, IL, USA). For PRT, both the objects used were identical during the trial phase. However, during the testing phase, one of the objects was transferred to a new location in the PVC box. Thus, the time the rats spent at the new location was recorded using ANY-maze™ Video Tracking System (Stoelting Co., Wood Dale, IL, USA). All data obtained from NORT and PRT were expressed as recognition index (%) [43].

### 2.11. Fasting Blood Glucose Level

All rats were fasted for 12 h overnight with free access to water on PND 21 before fasting blood glucose (FBG) analysis. The following morning, the tails of all male offspring were pricked, and blood was sucked using a glucose strip. The FBG levels were recorded using a glucometer (Glucocard™ 01-mini, Arkray Factory, Inc., Kyoto, Japan) [44].

### 2.12. Postmortem Fat Percentage (%) Analysis

Upon sacrificing the rats on PND 21, the body fat (brown adipose tissue, retroperitoneal fat, visceral fat, and gonadal fat) was extracted from the rats, and their weights were measured. The weights were determined based on 100 g of body weight [45].

### 2.13. Insulin Level

Upon sacrificing the rats on PND 21, blood samples (4–5 mL) were collected using a heparin tube, while the hypothalamus was harvested and stored at −80 °C for further analysis. The blood samples were then subjected to centrifugation at 3500 rpm for 15 min to obtain the plasma. The plasma insulin level was then measured using commercial rat insulin ELISA kit (Shibayagi Co., Ltd., Gunma, Japan) [46].

### 2.14. Lipid Profile

The level of low-density lipoprotein (LDH), high-density lipoprotein (HDL), triglycerides, and total cholesterol in blood were measured using a diagnostic reagent test kit (Roche, Germany) using Hitachi Automatic Analyzer 902 (Tokyo, Japan) [47].

### 2.15. Antioxidant Parameter

The hypothalamus was defrosted at room temperature, minced into small pieces, and diluted with 1:15 w:v of phosphate buffer saline (PBS). The samples were then homogenised (Omni TH, Omni International, Kennesaw, GA, USA) together with protease inhibitor and butylated hydroxytoluene followed by sonication three times, each lasting about 20 s using ultrasonic cell disrupter (UP 400S, Hielscher, Teltow, Germany). The final homogenates were then subjected to centrifugation at 5000× *g* for 20 min [48]. The supernatant was then collected, and the concentrations of ferric reducing ability of plasma (FRAP) in the hypothalamus and plasma were analysed using double-antibody sandwich enzyme-linked immunosorbent assay ELISA kits (Cayman Chemical Company, Ann Arbor, MI, USA) [49]. A similar protocol was adapted to measure the hypothalamus and plasma concentrations of glutathione (GSH) using glutathione ELISA assay kits (Cayman Chemical Company, Ann Arbor, MI, USA).

### 2.16. Statistical Analysis

Data were analysed using SPSS version 27.0. Normality tests were performed on all obtained data to ensure normal distribution of the results. All data were expressed as mean ± standard error of the mean (SEM). To test for significant differences among the six groups, a one-way ANOVA was conducted. If the resulting *p*-value was less than 0.05, a Bonferroni correction test was employed to adjust the significance level and account for the increased risk of false positives associated with multiple comparisons. Because there were six groups, the adjusted significance level was set to *p* < 0.0083 (0.05 divided by 6) to maintain an overall family-wise error rate of 0.05. This allowed for a more accurate determination of statistically significant differences among the groups. Different letters in the figures and tables indicate a significant difference.

## 3. Results

### 3.1. Body Mass Index (BMI), Lee Index, and Abdominal Circumference of Male Offspring on PND 21

**Figure 1:**
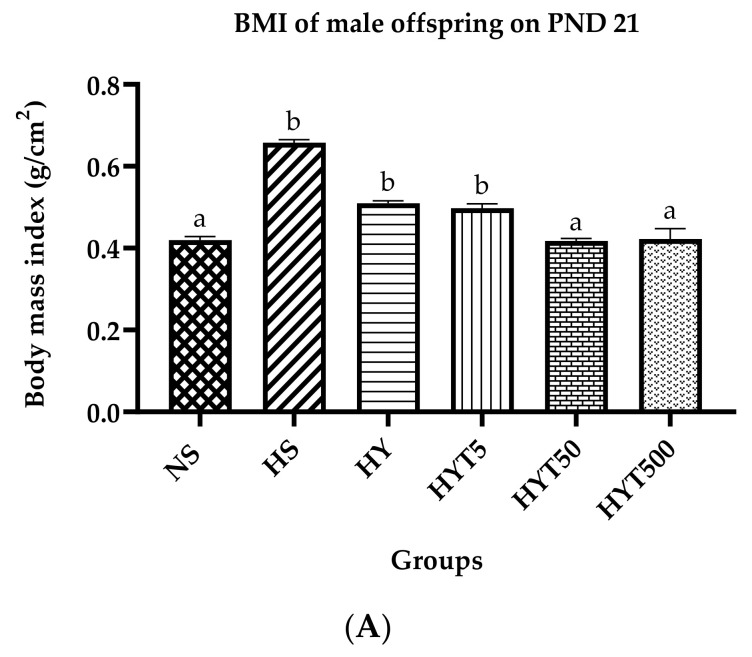

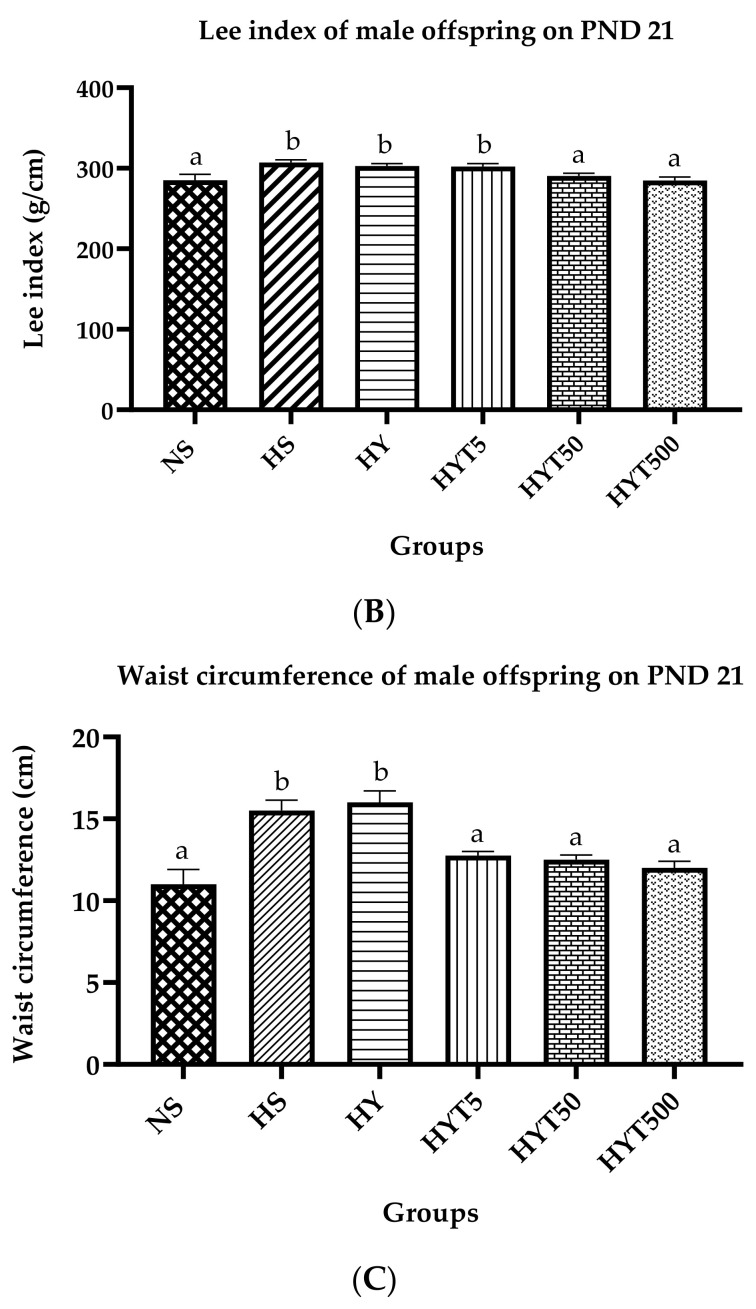
(**A**). BMI of male offspring on PND 21. (**B**). Lee index of male offspring on PND 21. (**C**). A waist circumference of male offspring on PND 21. Different letters indicates a significant difference at *p* < 0.05.

Figure 1A shows the BMI of male offspring on PND 21. The data show that the BMI of male offspring in HS, HY, and HYT5 is significantly higher compared to NS. There is no significant difference in the BMI of HYT50 and HYT500 compared to NS. As shown in Figure 1B, the Lee index of male offspring in HS, HY, and HYT5 is significantly higher compared to NS with a value of more than 310 g. There is no significant difference in the Lee index of HYT50 and HYT500 compared to NS. As shown in Figure 1C, the waist circumference of male offspring in HS, HY, and HYT5 is significantly higher compared to NS, while there is no significant difference in the waist circumference of HYT5, HYT50, and HYT500 compared to NS.

### 3.2. Fasting Blood Glucose in Male Offspring on PND 21

**Figure 2:**
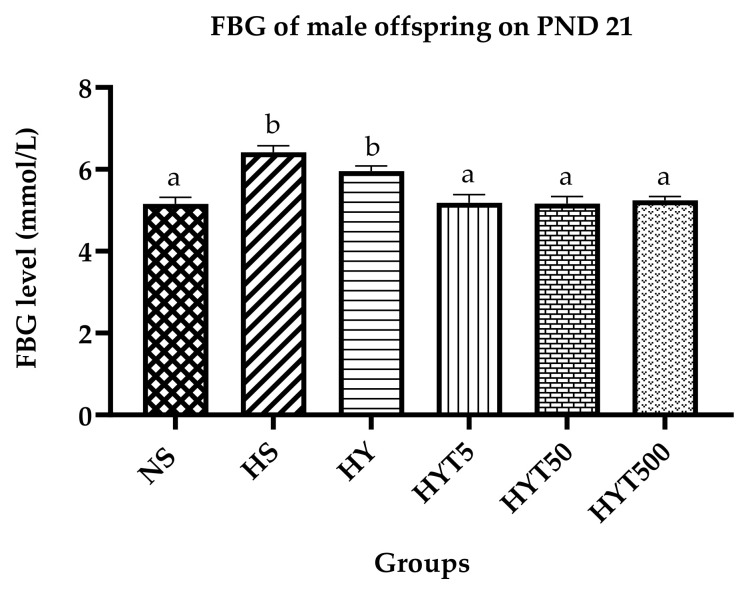
Fasting blood glucose (FBG) of male offspring on PND 21. Different letters indicates a significant difference at *p* < 0.05.

Figure 2 shows the FBG level of male offspring on PND 21. The data show that the FBG level of male offspring in HS, HY, and HYT5 is significantly higher compared to NS, while there is no significant difference in the plasma FBG level of HYT5, HYT50, and HYT500 compared to NS.

### 3.3. Anxiety Test in Male Offspring on PND 21

**Figure 3:**
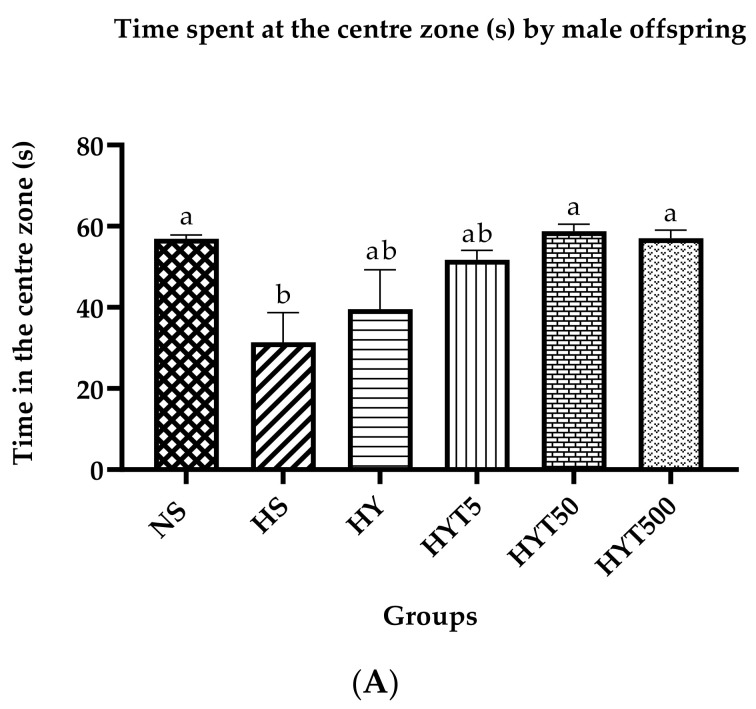

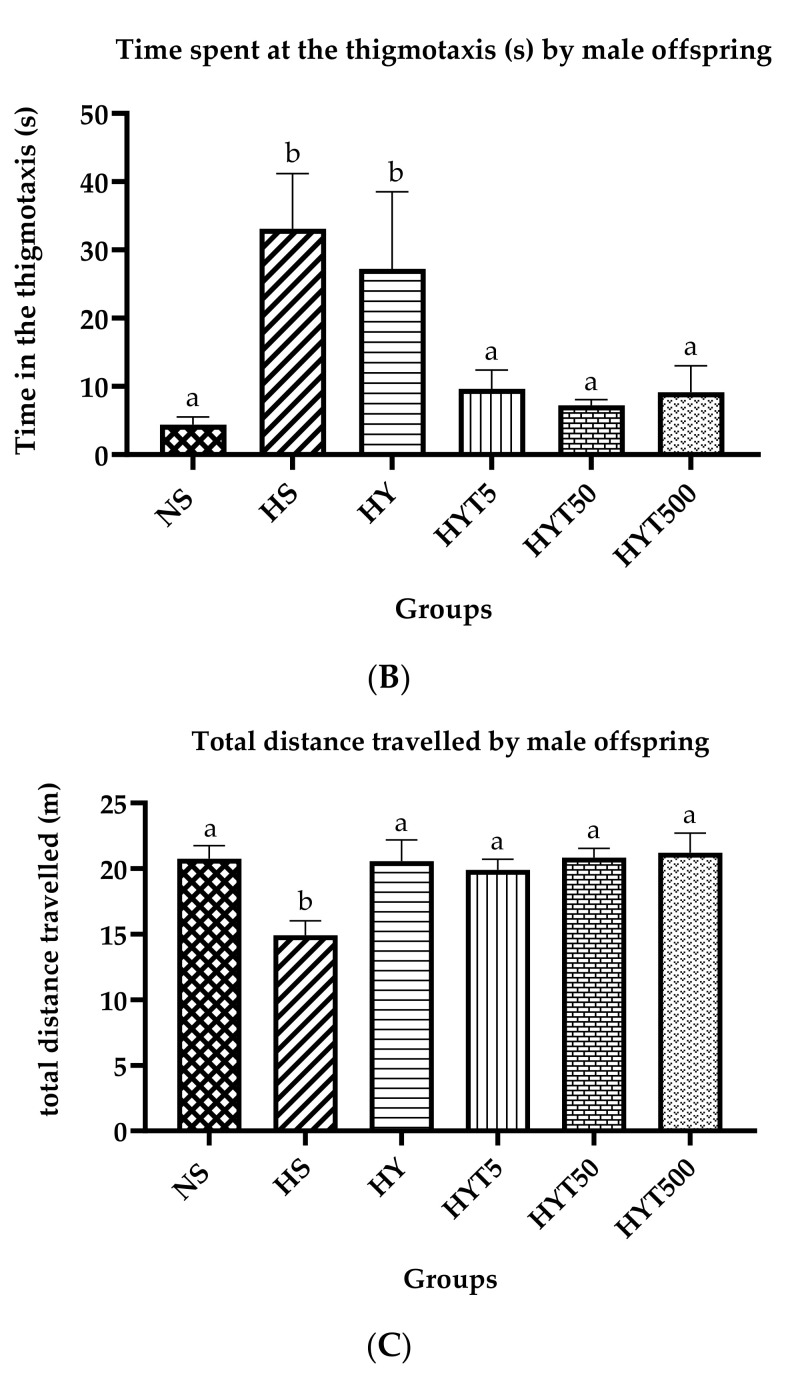
(**A**). Time spent at the central zone by male offspring in an open field test. (**B**). Time spent at thigmotaxis by male offspring in an open field test. (**C**). Total distance travelled in the central zone by male offspring in an open field test. Different letters indicates a significant difference at *p* < 0.05.

Figure 3A shows the time spent by male offspring in the central zone of the open field test (OFT). The data show that the male offspring in HS spend a significantly low duration of time compared to NS in the central zone. There is no significant difference in the duration of time spent by HY and HYT5 in the central zone compared to NS and HS. However, the time spent by HYT50 and HYT500 is similar to NS in the OFT. As shown in Figure 3B, the male offspring in HS and HY spend a significantly high duration of time compared to NS in the thigmotaxis. There is no significant difference in the duration of time spent by HYT5, HYT50, and HYT500 at the thigmotaxis compared to NS. Figure 3C shows the total distance travelled by male offspring in the central zone of the OFT. The data show that the total distance travelled by male offspring in HS is significantly lower compared to NS, whereas there is no significant difference in the total distance travelled by HY, HYT5, HYT50, and HYT500 in the central zone compared to NS.

### 3.4. Novel Object Recognition Test (NORT) and Place Recognition Test (PRT) in Male Offspring on PND 21

**Figure 4:**
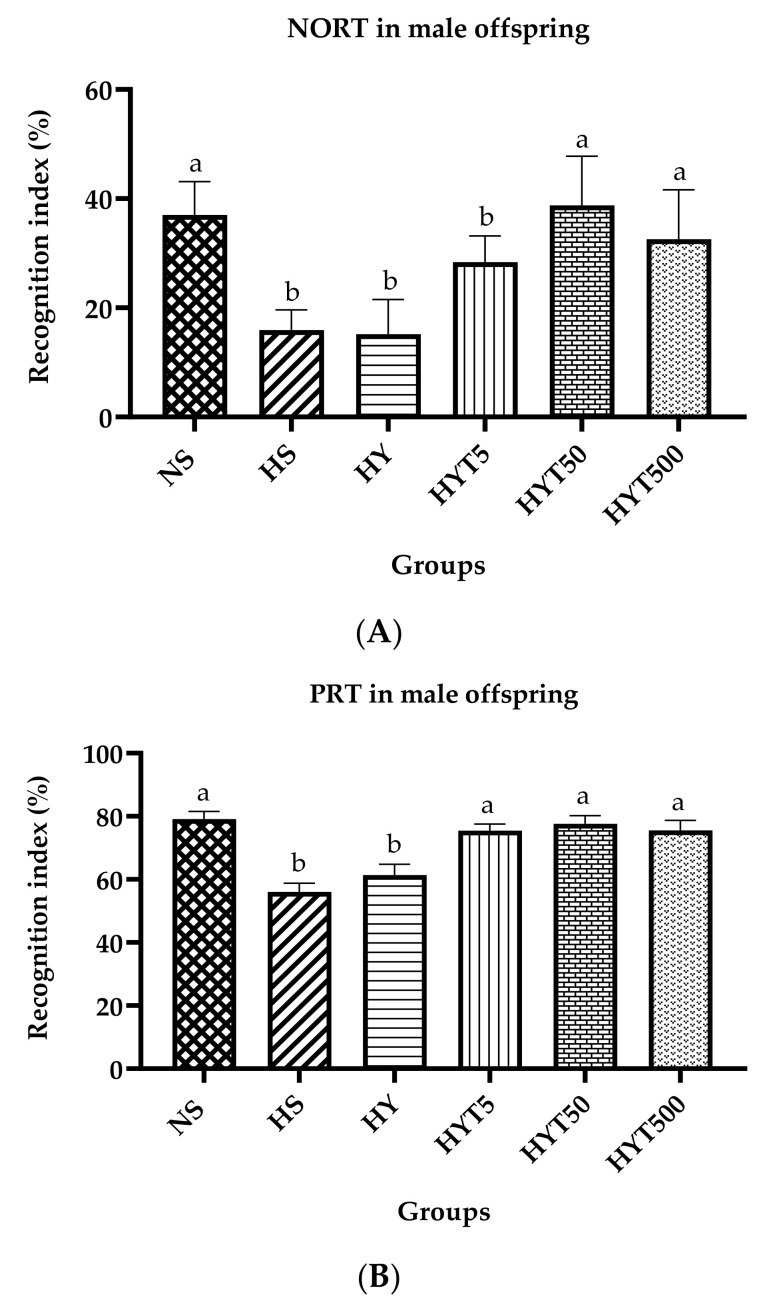
(**A**). Recognition index of male offspring in novel object recognition test (NORT). (**B**). Recognition index of male offspring in place recognition test (PRT). Different letters indicates a significant difference at *p* < 0.05.

Figure 4A,B show the recognition index (%) of male offspring in NORT and PRT on PND 21. As shown in Figure 4A, the recognition index of male offspring in HS, HY, and HYT5 is significantly lower compared to NS, whereas there is no significant difference in the recognition index of HYT50 and HYT500 compared to NS in NORT. The data in Figure 4B show that the recognition index of male offspring in HS and HY is significantly lower compared to NS, whereas there is no significant difference in the recognition index of HYT5, HYT50, and HYT500 compared to NS in PRT.

### 3.5. Fat Percentage (%) in Male Offspring on PND 21

Table 1 shows the fat percentage in male offspring on PND 21. The percentage of brown adipose tissue (BAT) and retroperitoneal white adipose tissue (RpWAT) and visceral and gonadal fat in male offspring of HS is significantly higher compared to NS. In male offspring of HY, the percentage of BAT shows no significant difference compared to HS. The fat percentage of RpWAT and visceral and gonadal fat in male offspring of HY is significantly lower compared to HS while significantly higher compared to NS. There is no significant difference in HYT5, HYT50, and HYT500 for BAT and RpWAT and visceral and gonadal fat compared to NS. The fat percentage of visceral and gonadal fat of male offspring in HYT50 and HYT500 shows no significant difference compared to HY. The RpWAT in male offspring of HYT50 shows no significant difference compared to HY.

### 3.6. Lipid Profile of Male Offspring on PND 21

**Figure 5:**
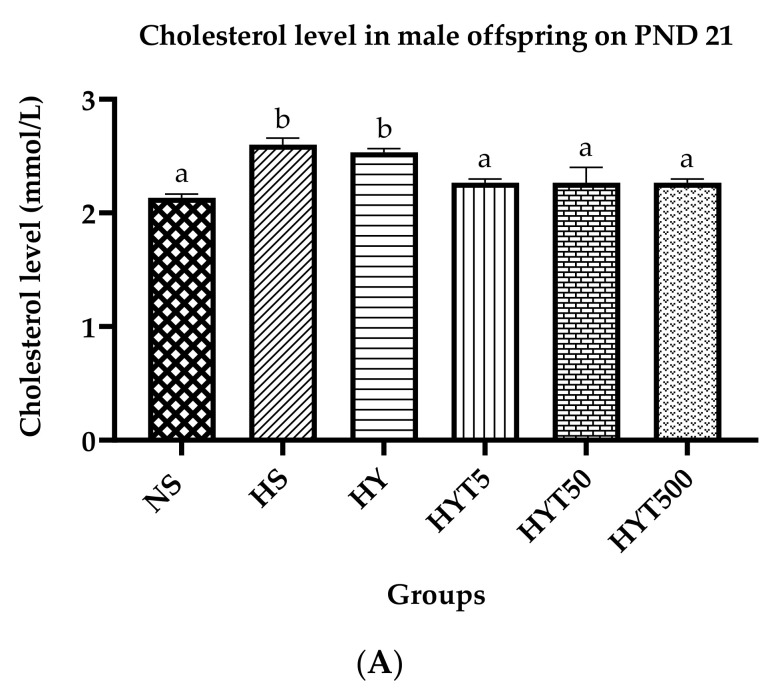

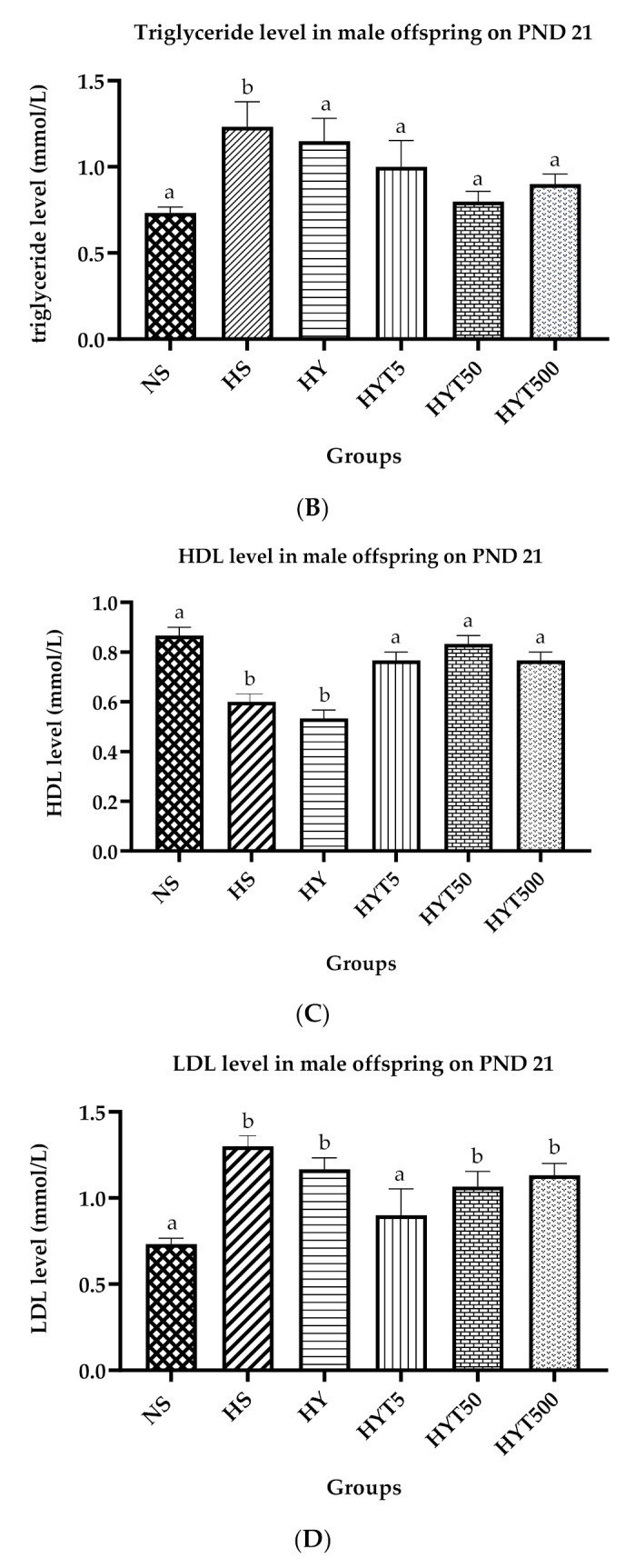
(**A**). Cholesterol levels in male offspring on PND 21. (**B**). Triglyceride levels in male offspring on PND 21. (**C**). HDL levels in male offspring on PND 21. (**D**). LDL levels in male offspring on PND 21. Different letters indicates a significant difference at *p* < 0.05.

Figure 5A–D show the lipid profile of male offspring on PND 21. The data show that the serum cholesterol levels of male offspring in the HY and HS groups are significantly higher compared to NS. There is no significant difference in the serum cholesterol levels of HYT5, HYT50, and HYT500 compared to NS. As shown in Figure 5B, the serum triglyceride level of male offspring in HS is significantly higher compared to NS. There is no significant difference in the serum triglyceride of HY, HYT5, HYT50, and HYT500 compared to NS. Meanwhile, the plasma HDL levels in the male offspring in HY and HS are significantly lower compared to NS. There is no significant difference in the plasma HDL levels of HYT5, HYT50, and HYT500 compared to NS. The data shown in Figure 5D indicate that the plasma LDL levels in the male offspring of HY and HS are significantly higher compared to NS. There is no significant difference in the plasma HDL levels of HYT5, HYT50, and HYT500 compared to NS.

### 3.7. Insulin Level in Male Offspring on PND 21

**Figure 6:**
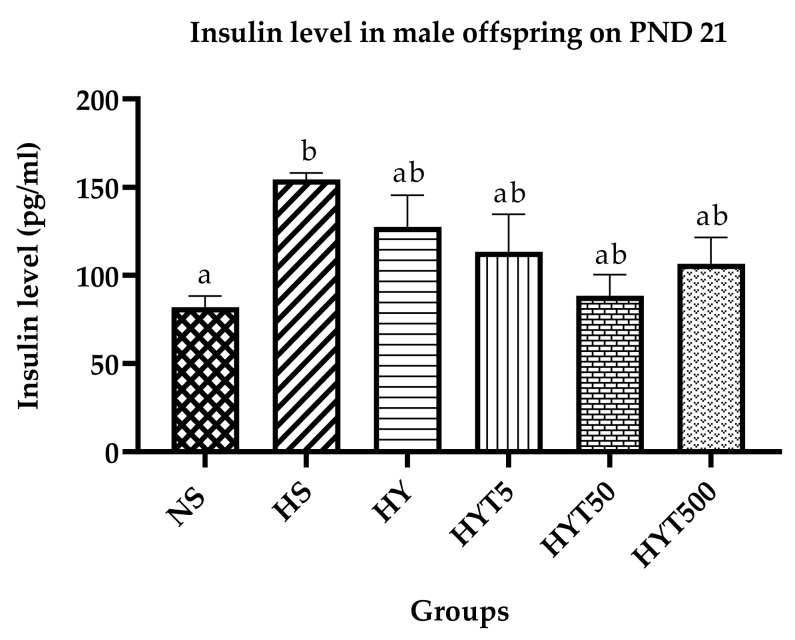
Insulin levels in male offspring on PND 21. Different letters indicates a significant difference at *p* < 0.05.

Figure 6 shows the plasma insulin levels of male offspring on PND 21. The plasma insulin level of male offspring in HS is significantly higher compared to NS, whereas there is no significant difference in the plasma insulin levels of HY, HYT5, HYT50, and HYT500 compared to NS.

### 3.8. Antioxidants Level in Serum and Hypothalamus of Male Offspring on PND 21

**Figure 7:**
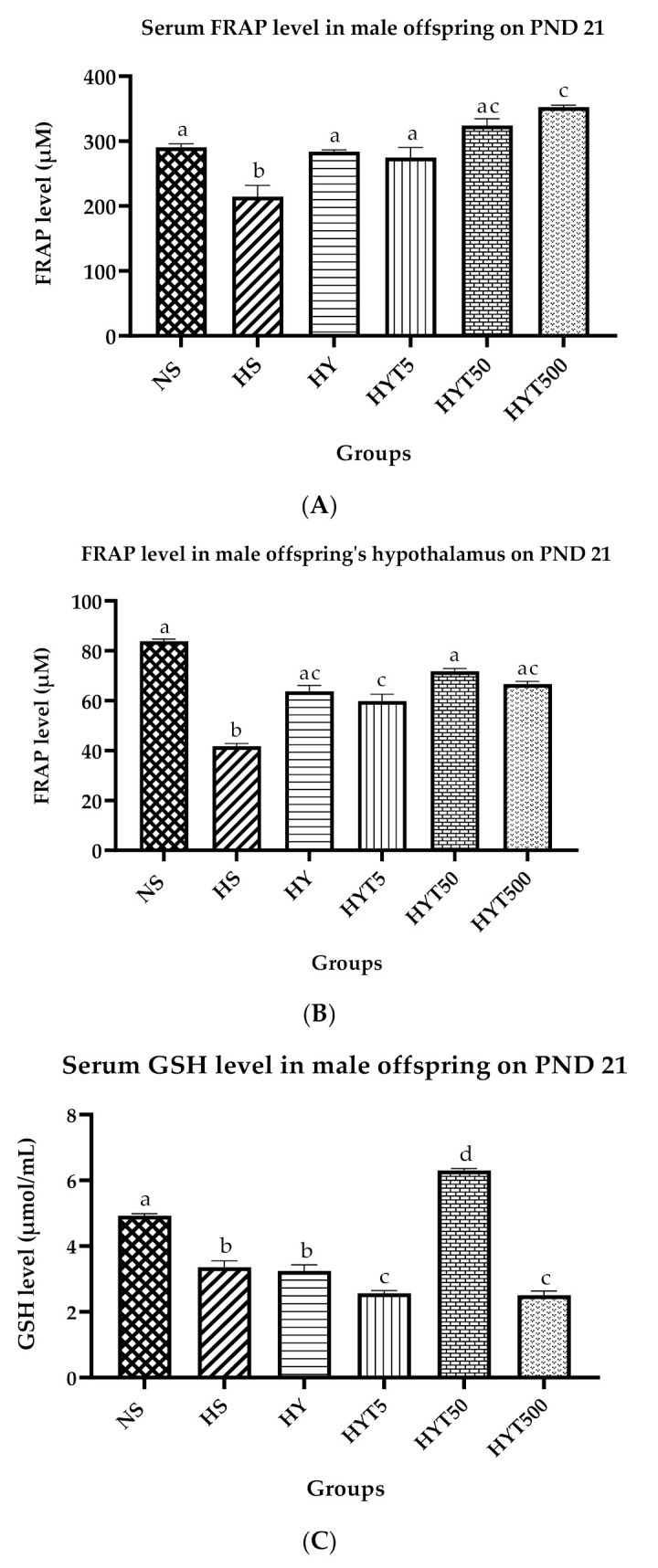

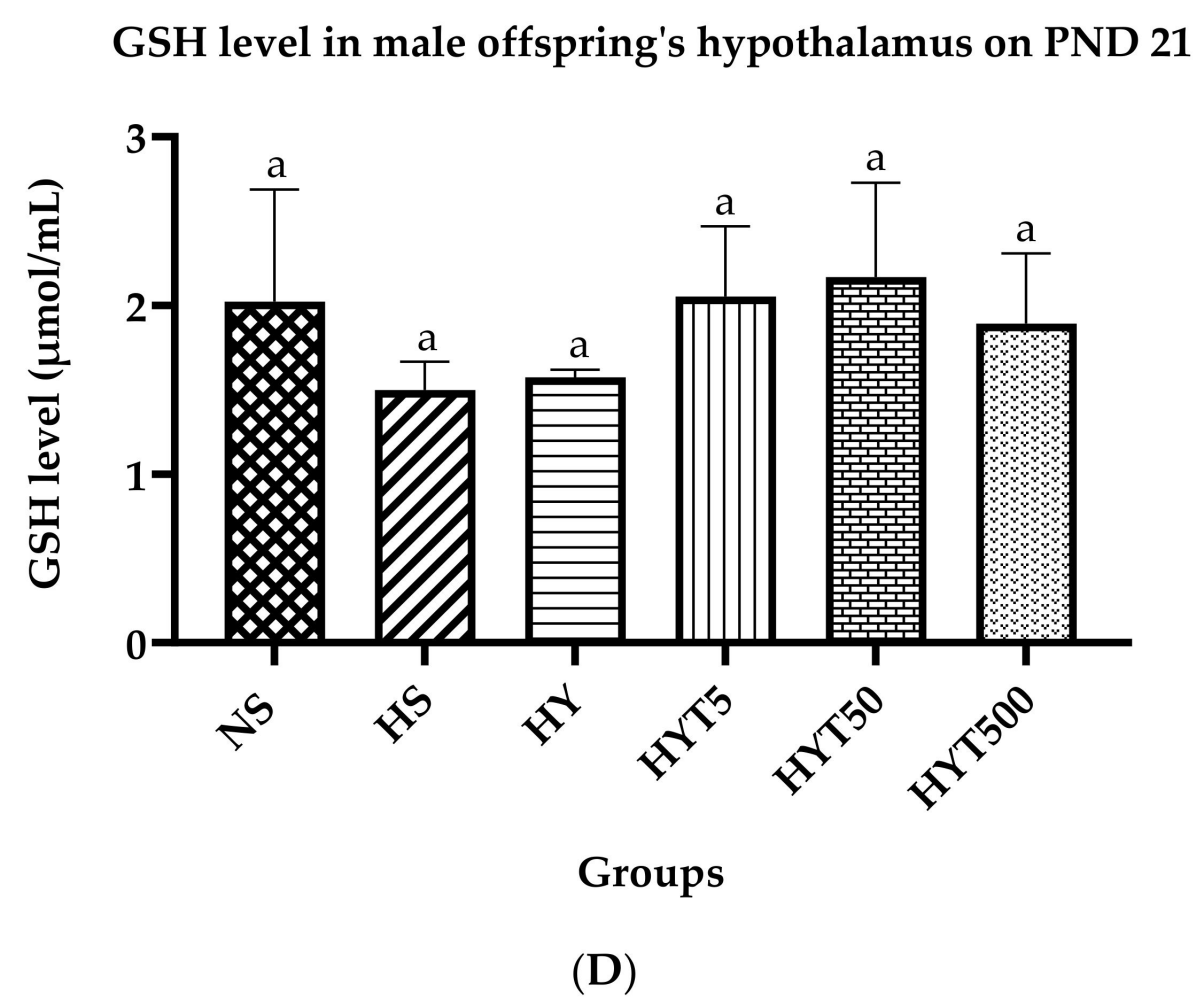
(**A**). The ferric reducing ability of plasma (FRAP) levels in male offspring’s serum on PND 21. (**B**). The ferric reducing ability of plasma (FRAP) levels in male offspring’s hypothalamus on PND 21. (**C**). Serum glutathione (GSH) levels in male offspring on PND 21. (**D**). Glutathione (GSH) levels in male offspring’s hypothalamus on PND 21. Different letters indicates a significant difference at *p* < 0.05.

Figure 7A–D show the antioxidant levels in the serum and hypothalamus of male offspring on PND 21. As shown in Figure 7A, the serum FRAP level is significantly lower in HS compared to NS. There is no significant difference in the serum FRAP levels of HY, HYT5, and HYT50 compared to NS. However, the serum FRAP level of HYT500 is significantly higher compared to NS, HS, HYT5, and HYT50, while there is no significant difference in the serum FRAP level of HYT50 compared to HYT500. As shown in Figure 7B, the FRAP levels in the hypothalamus are significantly low in HS and HYT5 compared to NS. There is no significant difference in the FRAP levels in the hypothalamus of HYT5, HYT50, and HYT500 compared to NS. The data in Figure 7C show the serum GSH levels. As shown in Figure 7C, the serum GSH levels are significantly lower in HS and HY compared to NS. Meanwhile, the serum GSH levels of HYT5 and HYT500 are significantly lower compared to HS, HY, and NS. However, the serum GSH level in HYT50 is significantly higher compared to HS, HY, HYT5, HYT500, and NS. The data in Figure 7D show the GSH level in the male offspring’s hypothalamus. As shown in Figure 7D, there is no significant difference among NS, HS, HY, HYT5, HYT50, and HYT500 in the hypothalamic GSH levels. However, the GSH level in the hypothalamus of male offspring in HYT50 shows a similar mean value to NS.

## 4. Discussion

Maternal obesity greatly affects the growth and behaviour of the child. Preliminary studies show that male offspring are more prone to glucose tolerance and increased levels of adiposity compared to female offspring due to overnutrition during the gestational period [50]. Similarly, offspring born to HFD-supplemented obese dams exhibited a high level of triglycerides, insulin, and expression of lipid genes [51]. Recent evidence claims that the offspring of obese mums possess 60% higher chances of developing ADHD and autism spectrum disorder (ASD) [52] and are more vulnerable to develop psychosocial difficulty [53]. Emerging evidence proves that nutritional intake during the gestational period greatly influences the behavioural changes of the offspring, particularly in hippocampal-dependent memory [54]. Thus, this study investigates the effects of early intervention of *Elateriospermum tapos* yoghurt in obese dams to mitigate intergenerational cognitive deficits and thigmotactic behaviour in male offspring, focusing on metabolic parameters and antioxidant changes in the hypothalamus. The key findings from this study show that the male offspring born to the HFD-supplemented group without any treatment (HS group) have a high BMI, Lee index, and waist circumference. They do possess a low level of recognition index in NORT and PRT as well as an increased level of thigmotactic behaviour with a reduced antioxidant profile in the hypothalamus. Their fat percentage in BAT and RpWAT and visceral, and gonadal fat is significantly high with an altered metabolic profile on PND 21. This outcome in this study proves the successful establishment of the intergenerational obese model in male offspring with a poor neurodevelopment condition. This result is similar to the study conducted by O’Reilly et al., 2013 [55], who noticed that an increase in BMI has a positive correlation with body fat content. Our result is in line with Oken et al., 2021, who demonstrated that maternal obesity influences poor memory, learning ability, and fetal brain development [56].

On the contrary, the male offspring of the yoghurt-supplemented obese dams (HY) show a slight reduction in body composition and metabolic profile and a slight improvement in memory compared to the HS group. Our result is similar to the study performed by Wiciński et al., 2020, as the child’s obese mum supplemented with probiotics shows normal body mass, metabolic profile, and inflammatory markers just as the negative control group [57], and the probiotic-containing *Lactobacillus* strain is able to restore cognitive decline in the offspring of HFD-supplemented dams [58]. This is because probiotics, such as yoghurt consumption during pregnancy, modulate gut microbiome composition and prevent gut dysbiosis in the foetus, thereby limiting the inheritance of the obesogenic gene in the child [59]. Similarly, the presence of proteins, iodine, and zinc in yoghurt enhances the memory function of the growing foetus [60]. However, the changes observed in the male offspring of dams administered with plain yoghurt (HY) are not as prominent as the male offspring of dams supplemented with *E. tapos*-integrated yoghurt (HY5, HY50, and HY500) in this study. This is because medicinal plant-integrated yoghurt exhibits more beneficial effects similar to the NS in the study. The outcome in HY50 and HY500 is almost similar to the NS group; however, male offspring HY50 exhibit a similar mean value as NS in all of the parameters accessed in this study.

Intriguingly, our data are similar to the previous study performed by Balan et al., 2021, who noticed that *E. tapos* extract was proven to inhibit the transgenerational inheritance of obesity in the female offspring [61] and ameliorate cognitive dysfunction in the F1 generation [62]. This is because *E. tapos* extract contains numerous bioactive compounds that have a molecular weight of ≤600 daltons [24] that could cross the placenta and blood–brain barrier to exhibit beneficial effects. In this context, *E. tapos* seed contains a high concentration of phenolic and flavonoids, which possess inhibitory activity on α-amylase, α-glucosidase, and pancreatic lipase [63]. The ability to inhibit pancreatic lipase by *E. tapos* prevents lipid absorption, thereby manifesting in a decreased level of fat content [64]. Meanwhile, the inhibitory activity of α-amylase prevents the absorption of carbohydrates and hydrolyses glucose into polysaccharides [65]. Resultantly, these changes may be evinced as decreased levels of cholesterol and glucose in the bloodstream. Similarly, the inhibition of α-glucosidase may reduce triglycerides and hyperinsulinaemic conditions [66]. Proportionately, such changes in the metabolic profile could be the underlying mechanism of male offspring belonging to HYT5. HYT50 and HYT500 show a gradual reduction in the BMI, Lee index, and waist circumference compared to the HY group in this study.

In the bargain, the presence of flavonoids, such as kaempferol and amentoflavone in *E. tapos* extract [67], is one of the reasons for the increased levels of GSH in the male offspring of HYT5, HY50, and HYT500 in the hypothalamus compared to the plain yoghurt-supplemented group (HY) and HS. This is because those flavonoids are strong antioxidants that exhibit protective effects against oxidative stress or inflammation via various signalling pathways [68,69]. Comparatively, a similar phenomenon is observed in the FRAP levels in the serum and hypothalamus of the *E. tapos* yoghurt-treated group, which proves that flavonoids could increase the level of antioxidants. Increased levels of antioxidants (FRAP and GSH) could neutralise free radicals and prevent inflammatory responses [70] that are released by an excessive level of adipose tissue in obesity. Thus, increased levels of GSH and FRAP in the hypothalamus may reverse memory decline and ease anxiety-like behaviour [71]. This is because GSH acts as a protective shield for neurons from stress disturbance [72], while FRAP prevents memory deficits by reversing the deprivation of neurotransmitters [73]. Hence, through the results obtained from this study, the hypothesis has been proven because the early intervention of *E. tapos* in obese dams prevents cognitive deficits and thigmotactic behaviour in male offspring via the modulation of the metabolic profile.

## 5. Conclusions

An HFD intake during pregnancy is one of the factors for maternal obesity, while maternal obesity is positively correlated with metabolic disturbance, cognitive decline, and anxiety-like behaviour in the offspring. The supplementation of *E. tapos* yoghurt during the gestational period to the HFD-fed obese dams has proven to mitigate intergenerational cognitive deficits and thigmotactic behaviour in male offspring via the modulation of the metabolic profile at the dose of 50 mg/kg/day.

## 6. Limitation

One of the limitations of this study could be that the outcomes from this study may not provide a complete understanding of how the intervention impacts female offspring. There could be significant differences in how the intervention affects the metabolic profiles and cognitive functions of male and female offspring. Thus, from the outcomes of this study, it may not be possible to generalise the findings to female offspring. This is because there may be gender-specific differences in response to the intervention or in the manifestation of cognitive deficits and thigmotactic behaviour. Additionally, the study does not investigate the long-term effects of the intervention on the health outcomes of the male offspring or subsequent generations. Therefore, further research should be conducted to confirm the results and to explore the effects of the intervention on other populations.

## Figures and Tables

**Table 1:** Fat percentage (%) in male offspring on PND 21.

Group/ Fat Tissue	BAT	RpWAT	Visceral	Gonadal
NS	0.11 ± 0.27 ^a^	0.23 ± 0.06 ^a^	0.48 ± 0.09 ^a^	0.49 ± 0.14 ^a^
HS	0.23 ± 0.02 ^b^	1.08 ± 0.07 ^b^	1.26 ± 0.05 ^b^	0.95 ± 0.13 ^b^
HY	0.22 ± 0.01 ^b^	1.04 ± 0.10 ^c^	0.88 ± 0.08 ^c^	0.78 ± 0.02 ^c^
HYT5	0.13 ± 0.18 ^a^	0.66 ± 0.09 ^a^	0.71 ± 0.03 ^ac^	0.75 ± 0.02 ^ac^
HYT50	0.12 ± 0.02 ^a^	0.55 ± 0.04 ^ac^	0.62 ± 0.06 ^ac^	0.61 ± 0.05 ^ac^
HYT500	0.14 ± 0.02 ^a^	0.61 ± 0.09 ^a^	0.59 ± 0.07 ^ac^	0.60 ± 0.03 ^ac^

Different letters indicates a significant difference at *p* < 0.05.

## Data Availability

The dataset generated during and/or analysed during the current study is available from the corresponding author upon reasonable request.
